# Knockdown of long noncoding RNA HUMT inhibits the proliferation and metastasis by regulating miR-455-5p/LRP4 axis in hepatocellular carcinoma

**DOI:** 10.1080/21655979.2022.2051841

**Published:** 2022-03-16

**Authors:** Xianzhi Zou, Peng Sun, Hui Xie, Lu Fan, Kun Ding, Jiyang Wang, Yang Li

**Affiliations:** a Department of Medical Interventional Oncology, Yantai Qishan Hospital, Yantai, Shandong, China; b Department of Medical Gastroenterology, Yantai Qishan Hospital, Yantai, Shandong, China; c Department of Internal Medicine, Yantai Qishan Hospital, Yantai, Shandong, China; d Department of Liver Diseases, Yantai Qishan Hospital, Yantai, Shandong, China; e Department of Physical Examination Center, The Second Affiliated Hospital of Shandong University of Chinese Medicine, Jinan, Shandong, China; f General Medical Department, Shanxi Bethune Hospital, Shanxi Academy of Medical Sciences, Tongji Shanxi Hospital, Third Hospital of Shanxi Medical University, Taiyuan, Shanxi, China; g Tongji Hospital, Tongji Medical College, Huazhong University of Science and Technology, Wuhan, Hubei, China

**Keywords:** hepatic cancer, lncRNA HUMT, miR-455-5p, LRP4

## Abstract

The present study aimed at investigating the effects and mechanism of long noncoding RNA highly upregulated in metastatic triple-negative breast cancer lymph node (lncRNA HUMT) in hepatocellular carcinoma (HCC). Quantitative real-time polymerase chain reaction was used to assess the expression of HUMT, microRNA (miR)-455-5p, and low-density lipoprotein receptor-related protein 4 (LRP4) in HCC tissues. Colony forming and 5-ethynyl-2′-deoxyuridine assays were performed to assess cell proliferation. Transwell assay was performed to measure cell migration and invasion. Cell cycle distribution was assessed using flow cytometry. The protein expression of LRP4, proliferating cell nuclear antigen (PCNA), matrix metallopeptidase 2 (MMP-2), and MMP-9 was detected using western blot. Luciferase reporter assay and RNA immunoprecipitation assay was used to confirm the target association between miR-455-5p and HUMT or LRP4. In our study, the level of HUMT was enhanced in HCC tissues and cells. Cell proliferation, invasion, and migration in HCC cells were repressed by knockdown of HUMT, and knockdown of HUMT arrested cells in G1 phase and decreased the levels of PCNA, MMP-2, and MMP-9. MiR-455-5p was a target of HUMT. Lowexpression of miR-455-5p reversed the inhibitive influence on HCC cells induced by of HUMT silencing. LRP4 was a target of miR-455-5p and was negatively regulated by miR-455-5p. In addition, LRP4 expression was positively modified by HUMT, and LRP4 inhibited the inhibitory effects on HCC cells induced by HUMT silencing. In conclusion, HCC cell proliferation, invasion, and migration were restrained by knockdown of HUMT, which was related to the miR-455-5p/LRP4 axis.

## Graphical Abstract

**Figure:**
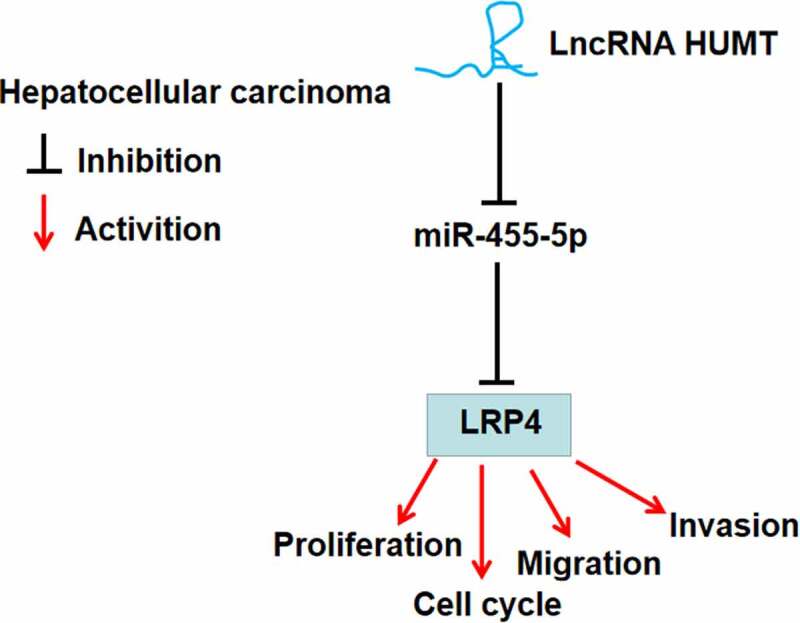

## Introduction

Hepatocellular carcinoma (HCC) has been listed as the second leading cause of death and the fifth leading cause of all cancer cases, seriously endangering human life and health [1]. In the past 20 years, the incidence rate of HCC has rapidly increased year by year because of the impacts of economic development and other factors, and it occurs in all age groups [2,3]. At present, the treatment of HCC is limited, and the commonly used methods are tumor ablation, liver resection and hepatic transplantation [4]. In addition, transarterial chemical embolization and sorafenib are regarded as palliative treatments [5]. Despite the rapid development of treatment methods in the past few decades, the overall long-term survival rate is still very low in HCC [6]. Moreover, due to the complex pathogenesis and the high recurrence rate after resection, there is still no clear and effective treatment method of HCC [7]. Thus there is an urgent need for better solutions.

Long noncoding RNAs (lncRNAs) consist of more than 200 nucleotides and act as crucial effects in a variety of cancers [^8–11^]. It is reported that lncRNAs take part in various biological processes, including proliferation, metabolism, immunity, differentiation, and migration [12,13]. Imbalance of lncRNAs was found in HCC [14,15]. Liu et al. found that the expression of lncRNA cytoskeleton regulator RNA (CYTOR) was upregulated in HCC tumor tissues, and knockdown of CYTOR inhibited HCC progression [16]. Wu et al. [17] reported that high-expression of CCAAT/enhancer binding protein-alpha antisense 1 facilitated HCC progression. It has revealed that lncRNA focally amplified lncRNA on chromosome 1 facilitated HCC cell proliferation and migration by regulating miR-1236 [18]. LncRNA highly upregulated in metastatic triple-negative breast cancer lymph node (HUMT), as one of the member of lncRNAs, is reported to accelerate metastasis in triple-negative breast cancer [19]. Nevertheless, the mechanism of HUMT in the development of HCC remains indistinct.

MicroRNAs (miRNAs) consist of 18–25 nucleotides and can regulate target genes by complementary binding to their 3′-UTR [20]. Studies have shown that miRNAs are related to cell proliferation, differentiation, apoptosis, lipid metabolism, inflammation, immune responses, and oncogenesis [^21–23^]. Recently, miR-455-5p has been reported to participate in multiple disorders. For example, miR-455-5p had the neuroprotective effects on spinal cord ischemia reperfusion injury by inhibiting apoptosis of neurons [24]. MiR-455-5p inhibits vascular smooth muscle cell proliferation and migration [25]. Previous studies have revealed that miRNAs help to seek diagnostic biomarker, and offering new targets for HCC [^26–28^]. Through targeting insulin-like growth factor-1 receptor, miR-455-5p restrained the proliferation and invasion of HCC cells [29]. Low-density lipoprotein receptor-related protein 4 (LRP4), a member of the LDL receptor family, contains a large extracellular domain, a few EGF-like domains, a transmembrane domain, and a small intracellular domain [30]. It is reported that LRP4 plays an important role in formation of the neuromuscular junction [31]. LRP4 induces extracellular matrix productions and promotes chondrocyte differentiation [32]. Mao et al. found that LRP4 promoted gastric cancer cell migration and invasion, and high expression of LRP4 was correlated with an unfavorable prognosis in GC patients [33]. However, the mechanism of miR-455-5p and LRP4 in HCC is unclear.

We hypothesized that lncRNA HUMT played an important role in HCC progression. This study aimed to investigate the function of HUMT in HCC and the specific mechanism of regulating miR-455-5p/ LRP4 axis.

## Materials and methods

### Bioinformatics analysis

To select the candidate lncRNAs involved in HCC, the lncRNAs in The Cancer Genome Atlas (TCGA) database were mapped on heatmap. Based on TCGA database, the levels of HUMT, miR-455-5p, and LRP4 was obtained using the DESeq2 R package [34]. False-discovery rate (FDR) method was used to calculate the adjusted P-values, absolute log2-fold change of >1 and FDR of < 0.05 were set as the cutoff. The overall survival of HUMT, miR-455-5p, and LRP4 in HCC patients was analyzed using the Kaplan–Meier curve method. Log rank test was performed to compare survival times between two groups (high and low).

### Patient samples

The study obtained the approval of the Ethics Committee of Yantai Qishan Hospital (approval no. 20200510001). Every patient or every patient’s guardian signed the ‘informed written consent’. The levels of HUMT, miR-455-5p, and LRP4 were determined in 30 pairs HCC tissues and matched to non-cancer tissues.

### Cell cultivation

From the Nanjing Cobioer Biosciences Co., LTD (Nanjing, China), human HCC cell lines (HCCLM3, MHCC97L, MHCC97H, and Huh7) were acquired. Dulbecco’s modification of Eagle’s medium (Sigma-Aldrich, USA) supplemented with 10% fetal bovine serum (FBS; HyClone, USA) and 1% penicillin/ streptomycin (Gibco, USA) was used to maintain these HCC cells. From American Type Culture Collection (Manassas, VA, USA), the human liver cell line (THLE-2) was acquired. THLE-2 cells were cultured in bronchial epithelial cell basal medium supplemented with 5 ng/mL epidermal growth factor, 70 ng/mL phosphoethanolamine, and 10% FBS at 37°C in a humidified incubator containing 5% CO_2_.

### Cell transfection

GeneCopoeia Co., Ltd. (Guangzhou, China) synthetized small interferring (si) RNA-HUMT#1, siRNA-HUMT#2, siRNA-negative control (si-NC), miR-455-5p mimic, mimic NC, inhibitor NC, miR-455-5p inhibitor, pcDNA3.1-LRP4 and pcDNA3.1-NC. HCCLM3 and MHCC97H cells were transfected with si-HUMT#1, si-HUMT#2, si-NC using lipofectamine 2000 (Invitrogen, USA). Meanwhile, HCCLM3 and MHCC97H cells transfected with si-HUMT#1 were cotransfected with inhibitor NC, miR-455-5p inhibitor, pcDNA3.1-LRP4, and pcDNA3.1. After 48 h, quantitative real-time polymerase chain reaction (qRT-PCR) was used to assess the transfection efficiency.

### qRT-PCR

TRIzol (Invitrogen, USA) was applied to extract total RNA. PrimeScript RT Reagent Kit (Takara, Japan) was applied to perform reverse transcription. SYBR Premix Ex Taq II (Takara) was performed to detect the levels of miR-455-5p, HUMT, and LRP4 in an ABI7300 real-time PCR machine (Thermo Fisher Scientific, USA). U6 and GAPDH served as internal control. The levels of HUMT, miR-455-5p, and LRP4 was analyzed using 2^−ΔΔCt^ method [35]. Table 1 displays the primers.

**Table 1.** The primers used in qRT-PCR

Name	Sequences
lncRNA HUMT forward lncRNA HUMT reverse miR-455-5p forward	5’-TACCCCTGGGCTTACCCTTT-3’ 5’-TGCCATTGGCCTTGAGGATT-3’ 5’- GCGGCGGGCACAAGAAATGATG-3’
miR-455-5p reverse	5’-ATCCAGTGCAGGGTCCGAGG-3’
U6 forward	5’-CGCTTCGGCAGCACATATAC-3’
U6 reverse	5’-TTCACGAATTTGCGTGTCAT-3’
LRP4 forward	5’-TTCCTCTTGATTTCAGGGAGGGC-3’
LRP4 reverse	5’-CAGACAGGAGGCAGATTCCC-3’
GAPDH forward	5’-CGGAGTCAACGGATTTGGTC-3’
GAPDH reverse	5’-TGGAATTTGCCATGGGTGGA-3’

### Fluorescence in situ hybridization (FISH) assay

HCCLM3 and MHCC97H cells (6 × 10^4^ cells/well) were seeded on a cover glass on the 24‐well culture. When the cell confluence reached about 80%, cells were fixed in 4% paraformaldehyde for 15 min. After being treated with protease K, glycine, and acylation reagent, cells were incubated with 250 µl prehybridization solution for 1 h at 42°C, and then incubated with hybridization solution containing lncRNA HUMT probes (RiboBio, China) overnight at 42°C. Next, the nucleus was stained with 4ʹ,6-diamidino-2-phenylindole 2hci (ab104139, 1:100, Abcam) for 5 min. Finally, cells were photographed with a fluorescence microscope (Olympus, Japan) [36].

### Colony formation assay

The transfected HCCLM3 and MHCC97H cells were plated into a 6-well plate. After incubation for 14 days, methanol (Sigma-Aldrich) was used to fix cells for 15 min and crystal violet (Sigma-Aldrich) was used to stain cells. Finally, the number of colony was assessed under a light microscope (Thermo Fisher Scientific) [37].

### 5-ethynyl-2′-deoxyuridine (EdU) assay

The transfected HCCLM3 and MHCC97H cells (1.5 × 10^5^ cells/well) were seeded into a 24-well plate. Next day, the cells were incubated with 50 µM EdU (RiboBio, China) for 2 h. Subsequently, 4% paraformaldehyde was used to fix cells for half an hour. Then cells were incubated with glycine (2 mg/ml), 0.5% Trion X-100, and stained with apollo® fluorescent dye and Hoeschst 33342. The inverted microscopy (Nikon, Japan) was applied to take the images [38].

### Cell cycle assessment

After transfection for 48 h, HCCLM3 and MHCC97H cells were fixed in ice-cold 70% ethanol for 4 h at 4°C. Next, cells were incubated with Propidium Iodide and RNase A (Beyotime Biotechnology) for 30 min at 37°C. Finally, cell cycle was evaluated using a flow cytometer (BD Biosciences, USA) [39].

### Cell invasion and migration assays

After transfection for 48 h, cell suspension was prepared. The upper chamber covered with (invasion assay) or without (migration assay) matrigel was inoculated with HCCLM3 and MHCC97H cells (1 × 10^5^), and the lower chamber was filled with 600 μl completely medium. The cells were cultivated at 37°C overnight, and then stained by 0.1% crystal violet. Finally, the microscope was applied to take the images of invaded or migrated cells [36].

### Western blot

Cells were lyzed with Radio-Immunoprecipitation Assay buffer (Aspen Biotechnology, China) to extract total proteins. The concentration of proteins was measured using Bicinchoninic acid Protein Assay kit (Thermo Fisher Scientific). Next, protein samples (30 μg) were loaded to 10% sodium dodecyl sulfate polyacrylamide gel electrophoresis and transferred onto polyvinylidene difluoride membranes (Millipore, USA). After being blocked in 5% skim milk for 1 h, the membranes were incubated with primary antibodies [LRP4, ab230188, Abcam, UK; proliferating cell nuclear antigen (PCNA), ab92552, Abcam; matrix metallopeptidase 2 (MMP-2), ab181286, Abcam; matrix metallopeptidase 9 (MMP-9), ab283575, Abcam; GAPDH, ab181602, Abcam] overnight at 4°C. After that, the membranes were incubated with horseradish peroxidase‐labeled goat anti‐rabbit secondary antibody (ab205718, Abcam) for 1 h at room temperature. Finally, the protein bands were visualized using an efficient chemiluminescenc (ECL) kit (Millipore), and the gray density of protein bands were evaluate using Image J software (National Institute Health, USA).

### Dual-luciferase reporter assay

When cells confluence reached 80%, miR-455-5p mimic, mimic NC, and the pmirGLO luciferase reporter gene vector with either HUMT-wild type (WT), LPR4-WT or HUMT-mutant type (MUT), LPR4-MUT was transfected into HCCLM3 and MHCC97H cells. The relative luciferase activities were detected after transfection for 48 h [40].

### RNA immunoprecipitation (RIP) assay

The Magna RIP RNA-Binding Protein Immunoprecipitation Kit (Millipore) was used to conduct RIP assay. Briefly, HCCLM3 and MHCC97H cells were lysed in a RIP-lysis buffer. Then, the RIP buffer containing magnetic beads conjugated with anti-Ago-2 (Millipore) or anti-IgG (Millipore) were added to the cell lysates and incubated for overnight at 4°C. Next, the magnetic beads were incubated with proteinase K buffer for 30 min. Finally, the enrichment of HUMT, miR-455-5p, and LRP4 in purified RNAs was examined by qRT-PCR [41].

### Statistical analyses

GraphPad Prism 7 (USA) was used to perform statistical analyses. Student’s t-test was used to analyze changes in experimental results between two groups. ANOVA with Dunnett t-test was performed for multiple comparisons. The relationship among miR-455-5p, HUMT, and LRP4 was analyzed using Pearson’s correlation analysis. Differences with P < 0.05 were considered as significant.

## Results

In the present study, we investigated the expression, function, and molecular mechanism of HUMT in HCC progression using bioinformatics, function-loss experiments. The findings showed that HUMT expression was upregulated in HCC tissues and cells, knockdown of HUMT inhibited HCC cell proliferation, migration, and invasion via regulating the miR-455-5p/LRP4 axis.

### LncRNA HUMT is upregulated in HCC tissues

**Figure 1.**
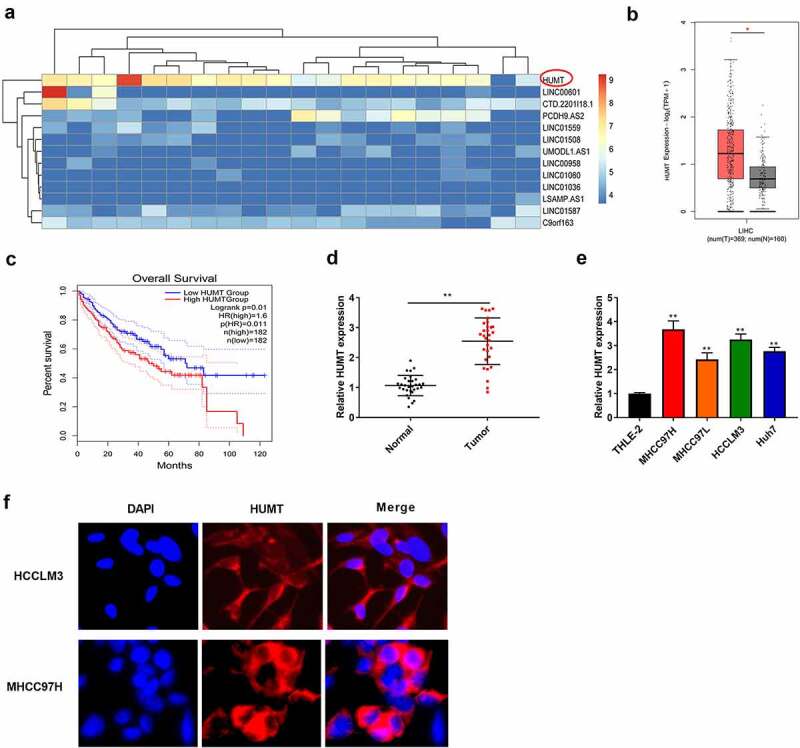
LncRNA HUMT expression was higher in HCC tissues. (a) The heatmap indicated the LncRNA expression in HCC. (b) TCGA database was used to analyze HUMT expression in HCC tissues. (c) According to TCGA database, the overall survival of HUMT in HCC patients was analyzed. (d) HUMT expression in normal and tumor tissues was tested by qRT-PCR (n = 30). (e) QRT-PCR was performed to assess HUMT expression in human HCC cell lines (HCCLM3, MHCC97L, MHCC97H, and Huh7) and human liver cell line (THLE-2). (f) The subcellular localization of HUMT was assessed using FISH assay **P < 0.01 vs. Normal group or THLE-2 cells.

Among the top differentially expressed lncRNAs between normal and HCC tissues in TCGA database, 2 aberrantly up-regulated and 11 down-regulated candidate lncRNAs were identified (Figure 1(a)). We chose HUMT as the candidate gene for HCC in the subsequent assays. Furthermore, to probe the expression level of HUMT in HCC patients, TCGA database was used. As presented in Figure 1(b), an obvious HUMT high expression was discovered in HCC patients relative to that in normal group. As presented in Figure 1(c), HUMT high expression was related to worse prognosis in HCC patients (P = 0.01). In addition, HUMT was higher in HCC tissues (Figure 1(d)). Figure 1(e) illustrates that HUMT expression was higher in HCC cell lines (MHCC97H, Huh7, HCCLM3, and MHCC97L). HCCLM3 and MHCC97L cell lines with higher HUMT expression were used in the subsequent assays. FISH assay was used to verify the subcellular localization of HUMT. The results showed that HUMT was mainly localized in the cytoplasm in HCCLM3 and MHCC97L cells (Figure 1(f)).

### The proliferation and metastasis of HCC cells is inhibited by knockdown of HUMT

**Figure 2.**
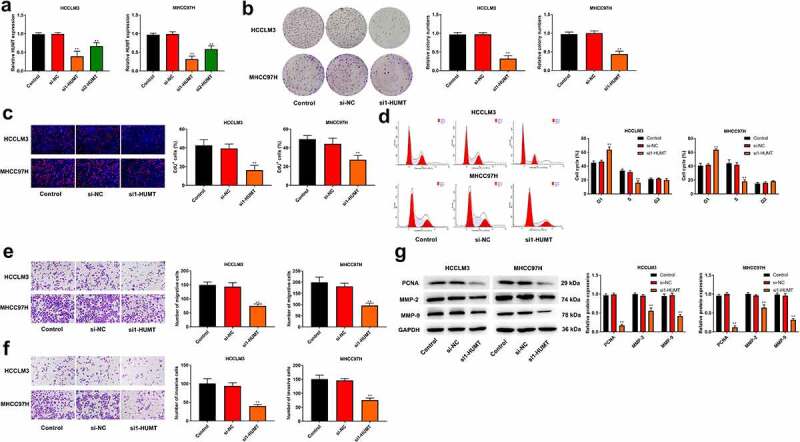
Knockdown of HUMT inhibited HCC cell proliferation and metastasis. (a) HUMT expression was assessed in HCCLM3 and MHCC97L cells using qRT-PCR. (b-c) Proliferation of HCCLM3 and MHCC97H cells was assessed using colony formation and EDU assays. (d) Cell cycle distribution in HCCLM3 and MHCC97H cells was assessed using flow cytometry. (e-f) HCCLM3 and MHCC97H cell migration and invasion were determined by transwell assay. (g) The protein expression of proliferatic and metastatic protein markers (PCNA, MMP-2, and MMP-9) in HCCLM3 and MHCC97H cells was detected using western blot.**P < 0.01 vs. Control or si-NC group.

Si-HUMT #1 and si-HUMT #2 were transfected into HCCLM3 and MHCC97L cells. Figure 2(a) illustrates that si1-HUMT and si2-HUMT reduced HUMT expression level in HCCLM3 and MHCC97L cells. Besides, the knockdown efficiency of si1-HUMT was higher than that in si2-HUMT, thus si1-HUMT was utilized in the subsequence assays. As shown in (Figure 2(b-c)), the ability of proliferation was significantly repressed by HUMT knockdown in HCCLM3 and MHCC97L cells. We then measured cell cycle distribution using flow cytometry. The results showed that knockdown of HUMT triggered G1 phase accumulation and decreased cell number of S phase in HCCLM3 and MHCC97L cells (Figure 2(d)). In addition, as presented in (Figure 2(e-f)), knockdown of HUMT decreased the number of migration and invasion in HCCLM3 and MHCC97L cells. The protein expression of proliferatic and metastatic protein markers (PCNA, MMP-2, and MMP-9) was detected using western blot. As expected, the results showed that knockdown of HUMT decreased the protein expression of PCNA, MMP-2, MMP-9 in HCCLM3 and MHCC97L cells (Figure 2(g)).

### MiR-455-5p is a target of HUMT in HCC

**Figure 3.**
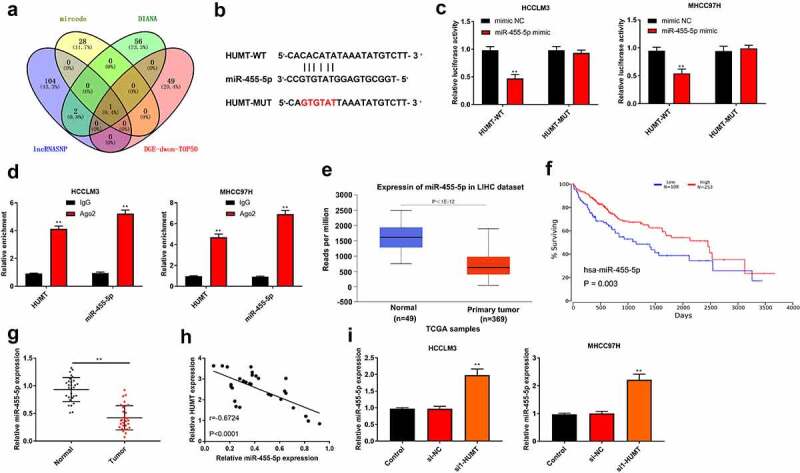
HUMT targets miR-455-5p in HCC. (a) One common miRNA (miR-455-5p) was gained from the intersection of venn diagram (mirode, DIANA, lncRNASNP, and DGE-down top50). (b) The binding sites of HUMT and miR-455-5p. (c) The targeting relationship between HUMT and miR-455-5p was verified by luciferase reporter assay in HCCLM3 and MHCC97H cells. (d) After anti-Ago2-mediated RIP assay, the expression of HUMT and miR-455-5p in HCCLM3 and MHCC97H cells was detected using qRT-PCR. (e)According to TCGA database, miR-455-5p expression in HCC was analyzed. (f) The overall survival of miR-455-5p in HCC patients from TCGA database. (g) QRT-PCR was performed to test miR-455-5p expression in normal and tumor tissues (n = 30). (h) The relationship between miR-455-5p and HUMT was analyzed by Pearson correlation analysis. (i) MiR-455-5p expression was examined in HCCLM3 and MHCC97H cells by qRT-PCR. **P < 0.01 vs. Normal, control, mimic NC, IgG or si-NC group.

To probe the mechanisms of HUMT in HCC, we first predicted its potential target genes. One common miRNA (miR-455-5p) was obtained from the intersection of mirode, DIANA, lncRNASNP, and DGE-down top50 (Figure 3(a)). Figure 3(b) displays the region of miR-455-5p targeted by HUMT. Figure 3(c) elucidates that the luciferase activity of HCCLM3 and MHCC97L cells containing HUMT 3’-UTR-WT instead of HUMT 3’-UTR-MUT was reduced by miR-455-5p mimic. Moreover, RIP assay showed that the enrichment of HUMT and miR-455-5p immunoprecipitated with Ago2 was increased compared with IgG precipitates in HCCLM3 and MHCC97L cells (Figure 3(d)). According to TCGA database, miR-455-5p was lowly expressed in HCC tissues, and low expression of miR-455-5p was related to worse survival (Figure 3(e-f)), manifesting that miR-455-5p was involved in HCC progression. As presented in Figure 3(g), miR-455-5p was downregulated in HCC tissues. Besides, there was a negative correlation between HUMT expression and miR-455-5p expression (r = −0.6724, P < 0.001, Figure 3(h)). Afterthat, we found that miR-455-5p expression was highly expressed in si1-HUMT group of HCCLM3 and MHCC97L cells (Figure 3(i)).

### HUMT regulates proliferation and metastasis through miR-455-5p in HCC

**Figure 4.**
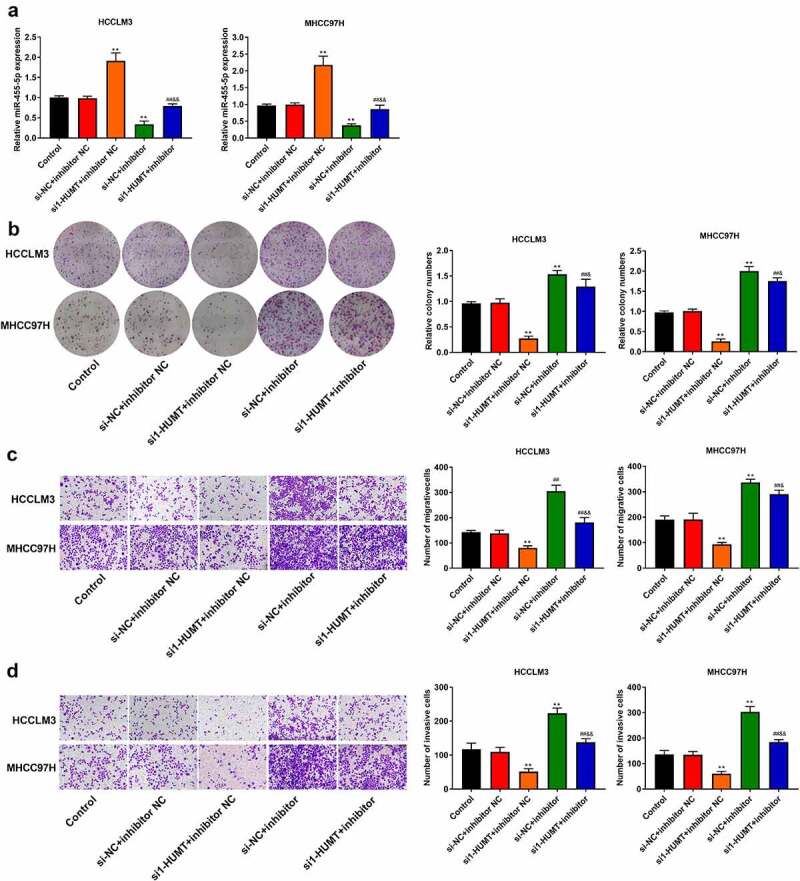
MiR-455-5p was related to the regulation of HUMT on HCC cell proliferation, migration, and invasion. (a) MiR-455-5p expression in HCCLM3 and MHCC97H cells was detected using qRT-PCR. (b) HCCLM3 and MHCC97H cell proliferation was detected using colony formation assay. (c-d) HCCLM3 and MHCC97H cell migration and invasion were determined by transwell assay. **P < 0.01 vs. Control or si-NC+inhibitor NC group. ^##^P < 0.01 vs. si1-HUMT+inhibitor NC group. ^&^P < 0.05 and ^&&^P < 0.01 vs. si-NC +inhibitor group.

To further probe the effects of miR-455-5p in the regulation of HUMT on HCC cell proliferation and motility, rescue assays were performed. Figure 4a shows that lowexpression of miR-455-5p weakened the promotive effect of HUMT ablation on miR-455-5p expression in HCCLM3 and MHCC97L cells. In addition, the down-regulation of miR-455-5p partly reversed the repressive effects of HUMT depletion on cell proliferation, migration, and invasion by in HCCLM3 and MHCC97L cells (Figure 4b-d).

### LRP4 is a target of miR-455-5p in HCC

**Figure 5.**
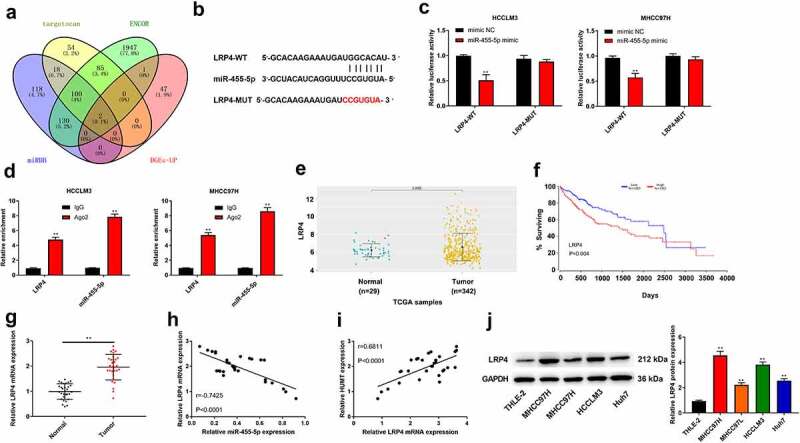
MiR-455-5p targets LRP4 in HCC. (a) The intersection of veen diagram (targetscan, ENCOR, miRDB, and DGE-up) showed 2 common genes. (b) The binding sites of miR-455-5p and LRP4. (c) The targeting relationship between LRP4 and miR-455-5p was confirmed by luciferase reporter assay in HCCLM3 and MHCC97H cells. (d) After anti-Ago2-mediated RIP assay, the expression of LRP4 and miR-455-5p in HCCLM3 and MHCC97H cells was detected using qRT-PCR. (e) TCGA database was used to analyze the level of LRP4 in HCCs. (f) The overall survival of LRP4 in HCC patients from TCGA database. (g) QRT-PCR assay was applied to test LRP4 expression in normal and tumor tissues (n = 30). (h, i) Pearson correlation analysis was performed to assess the relationship among HUMT, miR-455-5p and LRP4. (j) The protein expression of LRP4 in the human liver cell line (THLE-2) and HCC cell lines (HCCLM3, MHCC97L, MHCC97H, and Huh7) was detected using western blot. **P < 0.01 vs. Normal, mimic NC or THLE-2 group.

The potential target genes of miR-455-5p were predicted by bioinformatics analysis. The intersection of targetscan, ENCOR, miRDB, and DGE-up showed 2 common genes, and LRP4 was chosen for further research. The region of miR-455-5p targeted by LRP4 was displayed in Figure 5(b). Besides, Figure 5(c) elucidates that the luciferase activity of HCCLM3 and MHCC97L cells containing LRP4 3’-UTR-WT was weakened by miR-455-5p mimic. Moreover, RIP assay showed that the enrichment of LRP4 and miR-455-5p immunoprecipitated with Ago2 was increased compared with IgG precipitates in HCCLM3 and MHCC97L cells (Figure 5(d)). On the basis of the data acquired from TCGA database, LRP4 was highly expressed in HCC tissues (P = 0.0055), and high expression of LRP4 was related to worse survival (Figure 5(e-f)). Moreover, we found that LRP4 was higher in HCC tissues, and there was a negative correlation between LRP4 expression and miR-455-5p expression (Figure 5(g-h)). Nevertheless, as illustrated in Figure 5(i), there was a positive correlation between LRP4 expression and HUMT expression. In addition, the protein expression of LRP4 in the human liver cell line (THLE-2) and HCC cell lines (HCCLM3, MHCC97L, MHCC97H, and Huh7) was detected using western blot. The results showed that the protein expression of LRP4 in HCC cell lines was higher than that in THLE-2 line (Figure 5j).

### LRP4 overexpression reversed the inhibitory role of silencing HUMT on HCC cell proliferation and metastasis

**Figure 6.**
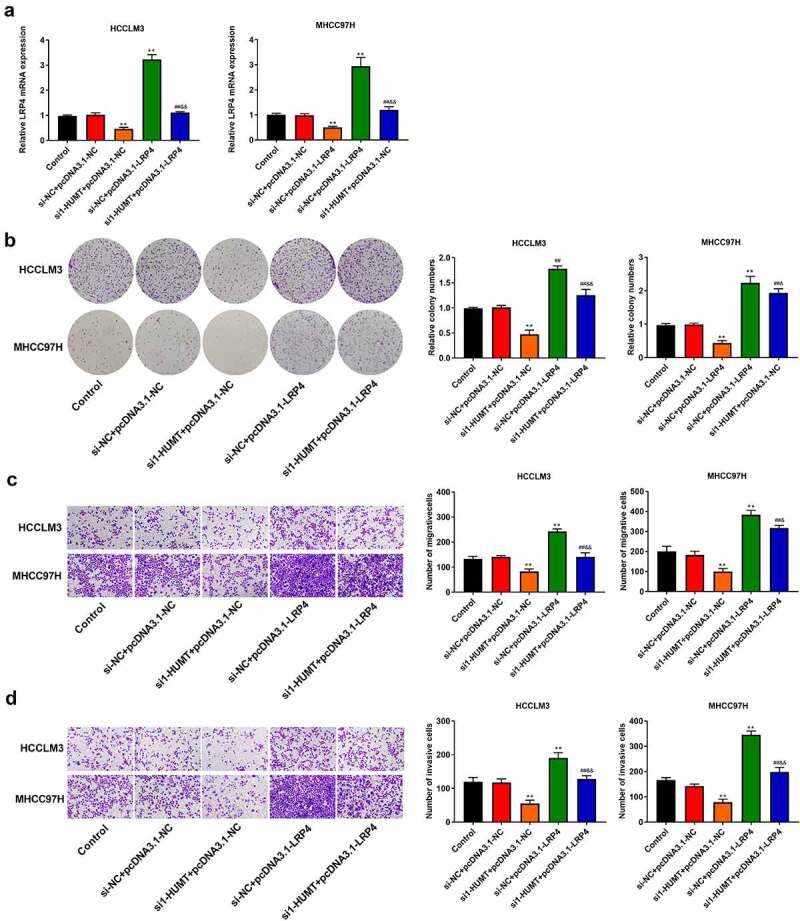
LRP4 overexpression inhibited the inhibitory role of silencing HUMT on HCC proliferation, migration, and invasion. (a) QRT-PCR assay was applied to detect LRP4 expression in HCCLM3 and MHCC97H cells. (b) Proliferation of HCCLM3 and MHCC97H cells was assessed using colony formation asssay. (c, d) The transwell assay was used to assess HCCLM3 and MHCC97H cell migration (c) and invasion (d). **P < 0.01 vs. Control and si-NC+pcDNA3.1-NC. ^##^P < 0.01 vs. si-HUMT+pcDNA3.1-NC. ^&^P < 0.05 and ^&&^P < 0.01 vs. si-NC + pcDNA3.1-LRP4.

We used qRT-PCR to test LRP4 expression in HCCLM3 and MHCC97H cells. Figure 6(a) shows that overexpression of LRP4 inhibited the repressive effect of HUMT knockdown on LRP4 expression. In addition, through colony formation and Transwell assays, we found that the LRP4 over-expression reversed the inhibitory effects of HUMT knockdown on cell proliferation, invasion, and migration in HCCLM3 and MHCC97H cells (Figure 6(b-d)).

## Discussion

LncRNAs are involved in growth, invasion, and migration in HCC [42,43]. As a highly dysregulated lncRNA, HUMT has been demonstrated to play a role in triple-negative breast cancer [19]. However, the mechanism related to HUMT in HCC progression remains unclear. We surveyed the influences of HUMT on HCC progression in this study. We found that HUMT was related to miR-455-5p/LRP4 axis in HCC.

We identified that HUMT expression was increased in HCC tissues and cells, and then explored the function and underling mechanisms. The findings indicated that knockdown of HUMT inhibited HCC cell proliferation and metastasis and arrested cells in G1 phase. Knockdown of HUMT decreased the protein expression of PCNA, MMP-2, MMP-9 in HCCLM3 and MHCC97L cells. Moreover, HUMT exerted its function through the miR-455-5p/LRP5 axis. Through protein kinase B/mammalian target of rapamycin/vascular endothelial growth factor signaling, HUMT exerted its function of proliferation and metastasis by recruiting Y-box binding protein 1 protein in triple-negative breast cancer [19]. Agreed with the above findings, our study confirmed that HUMT participated in cell proliferation and metastasis in HCC.

To figure out whether HUMT acts as a competing endogenous (ce) RNA in HCC, we performed bioinformatics analysis, RIP assay, and luciferase reporter assay. The results showed that miR-455-5p was a target of HUMT in HCC cells. Furthermore, miR-455-5p lowexpression reversed the repressive impact of HUMT knockdown on HCC cell proliferation and metastasis, indicating the roles of HUMT was involved in miR-455-5p. In addition, we searched and confirmed the target relationship between miR-455-5p and LRP4 by bioinformatics analysis and luciferase reporter assay. Functional analysis showed that cell proliferation, invasion, and migration were promoted by lowexpression of miR-455-5p in HCC cells, which is also consistent with former researches [29,44]. Under the regulation of miR-140-5p, LRP4 accelerated cell migration and invasion in gastric cancer [33]. In this study, LRP4 and HUMT have the binding sites of miR-455-5p, which supported that HUMT acted as a ceRNA. Our results also displayed the promoting effects of LRP4, revealed by abrogating the facilitative role of HUMT knockdown in HCC progression. Besides, LRP4 expression was regulated by HUMT and miR-455-5p, which indicated that HUMT could regulate LRP4 by sponging miR-455-5p in HCC.

## Conclusion

Taken together, HUMT expression was upregulated in HCC tissues and cells. HCC cell proliferation, invasion, and migration were inhibited by knockdown of HUMT, which was involved in the miR-455-5p/LRP4. These findings may provide novel clinical implications for HCC treatment.

## Data Availability

The datasets used and analyzed during the current study are available from the corresponding author on reasonable request.

## Highlight

1. LncRNA HUMT expression is upregulated in hepatocellular carcinoma tissues.
2. Knockdown of HUMT inhibits hepatocellular carcinoma cell growth and metastasis.
3. HUMT acts as a competing endogenous RNA of miR-455-5p to regulate LRP4 expression.
